# Developments in marine invertebrate primary culture reveal novel cell morphologies in the model bivalve *Crassostrea gigas*

**DOI:** 10.7717/peerj.9180

**Published:** 2020-06-01

**Authors:** Robert W.A. Potts, Alejandro P. Gutierrez, Yennifer Cortés-Araya, Ross D. Houston, Tim P. Bean

**Affiliations:** 1 The Roslin Institute and Royal (Dick) School of Veterinary Studies, University of Edinburgh, Edinburgh, United Kingdom; 2 Centre for Environment Fisheries and Aquaculture Science (Cefas) Weymouth Laboratory, Dorset, United Kingdom

**Keywords:** Primary cell culture, Pacific oyster, Tissue explant, Live cell imaging

## Abstract

Cell culture provides useful model systems used in a wide range of biological applications, but its utility in marine invertebrates is limited due to the lack of immortalised cell lines. Primary cell and tissue cultures are typically used but remain poorly characterised for oysters, which can cause issues with experimental consistency and reproducibility. Improvements to methods of repeatable isolation, culture, and characterisation of oyster cells and tissues are required to help address these issues. In the current study, systematic improvements have been developed to facilitate the culture of primary cells from adult Pacific oyster tissues and identify novel cell morphologies that have not been reported previously. Cultures analysed by light microscopy, qPCR, and live cell imaging demonstrated maintenance of live, metabolically active Pacific oyster cells for several weeks post-explant. Interestingly, whole hearts dissected from adult oysters were found to continue contracting rhythmically up to 8 weeks after being transferred to a tissue culture system. Mantle tissue explants were also actively moving in the culture system. These improvements in primary cell culture of bivalves may be beneficial for research in ecotoxicology, virology, immunology, and genetic resistance to disease.

## Introduction

Cell culture is a widely used model system for studying the biology of mammals, teleost fish and model invertebrates under controlled laboratory conditions. Cell culture methods for other classes, including non-model invertebrates, are less frequently used, less well optimised, and have proven difficult to develop into experimental systems (Cai & Zhang, 2014). Despite being a highly species rich phylum (Rosenberg, 2014), molluscan cell culture is especially limited, with only a single immortalised cell line (Bge) which was isolated from freshwater snail *Biomphalaria glabratra* embryos in 1976 (Hansen, 1976). Molluscan cell culture is otherwise limited to primary cultures, which typically reach senescence after a short number of passages, limiting their usefulness as models for experimental research (Yoshino, Bickham & Bayne, 2013). This typical of all marine mollusc species in which cell culture has been attempted, encompassing several commercially relevant oyster, mussel, scallop and clam species. The Pacific oyster *Crassostrea gigas* (Thunberg, 1973) is the most commercially important mollusc worldwide, and is frequently used in scientific research (FAO, 2018).

Primary molluscan cell cultures have been used for a wide range of studies including ecotoxicology (Ladhar-Chaabouni & Hamza-Chaffai, 2016), virology (Morga et al., 2017) and immunology (Dantas-Lima et al., 2012). Hemocytes are the most frequently used primary cells in Pacific oyster, as the method for establishing cultures is both relatively simple and well optimised (Renault et al., 2011). Hemocyte cultures have been used to study highly damaging diseases affecting commercial *C. gigas* production (Alfaro, Nguyen & Merien, 2018)*,* but cultures from other tissues have not been applied to this purpose, perhaps due to the difficulty of working with non-hemocyte cultures (Labreuche et al., 2006). Primary cell cultures do have some key advantages: they represent the original tissue more closely than cell lines; they are more similar to the in vivo state and exhibit physiological traits similar to whole animals. For this reason, they provide excellent model systems for studying the normal physiology and biochemistry of the animal, which may not be the case for an immortalised cell line (Alge et al., 2006; Pan et al., 2009). Primary cell cultures are also less susceptible to accidental cross contamination, which is a common problem associated with cell lines (Capes-Davis et al., 2010).

Development of molluscan cell cultures presents numerous challenges, which have contributed towards the lack of cell lines despite repeated efforts (Yoshino, Bickham & Bayne, 2013). This is especially relevant for marine invertebrate species, for which there are currently no cell lines available. The growth environment of marine invertebrates (including oysters) can be difficult to replicate in the laboratory, especially to conditions that stimulate proliferation, due to the highly variable physical and chemical conditions of the marine environment. Marine invertebrates, such as oysters, often operate an open body plan with the majority of individual organs coming into direct contact with seawater. This differs from some of the more advanced deuterostome species which have an homeostatic internal body environment. Therefore the media for mollusc cell culture needs to closely represent the marine environment, as well as the internal conditions of the animal. Various approaches have been used to replicate these conditions, including the use of filtered seawater or a mixture of salts to replicate seawater alongside conventional cell culture media (Chen & Wang, 1999; Daugavet & Blinova, 2015; Domart-Coulon et al., 1994; Le Deuff, Lipart & Renault, 1994). The presence of seawater adjacent to most tissues in marine invertebrates also means that tissues are regularly exposed to the wide community of microorganisms in the seawater. Indeed, this may be exacerbated by the filter-feeding nature of some marine invertebrates, such as the Pacific oyster. As a result, oysters that are used to establish cultures are regularly contaminated with marine fungi, protozoa, bacteria and viruses; and effective decontamination from marine microorganisms remains a major barrier to cell culture from oysters and other marine molluscs (Cai & Zhang, 2014). The lack of classification and understanding of certain crypto species which form common contaminants e.g., thraustochytrids (Rinkevich, 1999) means that there are no specific biocides. Further, as contamination with eukaryotic species is common, any treatment may also impact the oyster cells and hinder culture (Stacey, 2011). Molluscan cell culture is reliant on the use of both antibiotics and antifungal treatment, and previous studies have reported high frequency of contamination (Rinkevich, 1999; Yoshino, Bickham & Bayne, 2013). Primary cell cultures have previously been established either by dissociating tissues or allowing cells to migrate from tissue explants taken from the animal (Chen & Wang, 1999; Daugavet & Blinova, 2015; Wen, Kou & Chen, 1994). However, both of these approaches resulted in high rates of contamination (Rinkevich, 1999). A potential method to overcome this would be to treat large explants with universal biocides e.g., bleach at strong concentration. This would have a major negative impact on the surface cells and contaminants, but would have reduced impact on the cells at the centre of the explant.

Another problem common to molluscan primary cultures is the rapid onset of senescence. Primary cell cultures follow a consistent pattern of initial adherence and growth until senescence and or death (Daugavet & Blinova, 2015; Domart-Coulon et al., 1994; Le Deuff, Lipart & Renault, 1994; Renault, Flaujac & Le Deuff, 1995; Yoshino, Bickham & Bayne, 2013). Once cells reach senescence they are limited in their usefulness and as such delaying senescence is important to maximize primary culture utility. In turn, preventing senescence is an essential step in the generation of an immortalised cell line. Establishment of an immortalised bivalve cell line, or significant improvements in primary culture methods would be valuable to a wide range of fields. Despite frequent use and optimisation of methods, hemocyte cultures have limited potential for immortalisation as they are differentiated cells and have short lifespans. Therefore cultures of other cell types are likely to be crucial in further progressing this field (Fisher, 1986). Gill tissue has been suggested as the site of haematopoiesis in oysters and may therefore provide a greater potential for immortalisation (Jemaà et al., 2014). Literature regarding primary cell culture of gills is lacking and there is a high potential for contamination due to active filtering of seawater across the gills (Domart-Coulon et al., 2000; Gómez-Mendikute et al., 2005).

The primary aim of this study is to improve the state of the art for *C. gigas* primary cell and tissue culture. Key limitations that need improvement are (i) the limited range of culturable cell morphologies (ii) short lifespan of cultures (iii) scarcity of methodology for culturing cells from adductor muscle, gill and gonad (iv) high frequency of contamination. To help overcome the aforementioned issues, large explant cultures were investigated. Establishing new cultures with a large explant provides a greater quantity of cells, which are not stressed by the use of dissociation reagents, homogenisation or centrifugation. Furthermore, using a larger tissue explant method allows for a more vigorous decontamination process as cells within the tissue are protected from some of the negative effects of biocides by outer cells. This could be beneficial for understanding Pacific oyster biology, particularly characterisation and monitoring of destructive diseases. Furthermore, improved Pacific oyster cell culture can be used as a model for tissue culture across the broader group of marine invertebrates , where there is interest in other economically important areas of research such as pearl formation (Awaji & Suzuki, 1998) and biomineralisation (Daugavet & Blinova, 2015).

## Materials and methods

### Establishing cell cultures with a large explant method

Adult Pacific oysters were purchased from oyster farms in Northern England and Scotland between February and July 2019. On arrival at the laboratory, oysters were usually dissected immediately, or in some cases kept on ice for a maximum of 5 days. In order to extract hemolymph, the posterior-ventral join between the oyster valves we partially opened using a commercial oyster knife, without damaging the adductor muscle. Between 0 and 1,000 µl of hemolymph was aspirated from the adductor muscle of each oyster using a 21G 40 mm needle and 1 ml syringe, and placed immediately on ice. Oysters were then opened fully by removal of the right valve (see Fig. S1) and individual tissues (heart, gill, marginal mantle, adductor muscle, gonad and digestive gland) were dissected and pooled by tissue type in 50 ml falcon tubes with 25 ml of decontamination wash buffer. Hearts were dissected by cutting the connective-tissue which connects the atrium and ventricle to the rest of the animal, as such keeping the heart intact. The complete adductor muscle, including both smooth and striated muscle, was used (in the cases where the adductor split on the sagittal plane, and remained attached to both valves after opening, the largest of the two sections was used). Mantle and gill tissue were cut into sections (5 to 20 mm across by 20 to 40 mm long). Gonad and digestive gland explants were smaller (5 to 20 mm long) and were taken from the most clearly defined regions of each tissue; usually the dorsal end of the gonadal tissue and core sections of digestive gland. After dissection, the exterior surfaces of the falcon tubes containing the tissues were washed with 70% ethanol and transferred to laminar flow cell culture hood. Tissues were held in the initial decontamination wash for between 20 and 60 min then transferred into a second decontamination wash for 20 min, and a final decontamination wash for a further 20 min (60 to 100 min total). Tissues were moved from the final wash solution into Thermo Scientific™ Nunc™ (Massachusetts, United States) tissue culture (TC) plastic vessels with oyster culture media and incubated at 28 °C. Media was completely replaced after 48 h, which also served to remove any large debris from the media. Half the volume of media was refreshed and any further debris removed on days 5 and 8, and subsequently whenever there was an obvious colour change in the culture media. Cell cultures were examined regularly under microscope Zeiss Axiovert 40 inverted microscope and cultures with contamination were discarded. Explants were removed after a maximum of 2 weeks, unless there was obvious contraction visible (see Video S1) in which case the cultures were maintained for further study. Cells were cultured in a range of TC disposable plastic consumables including 6, 12, and 24 well plates and T25 flasks.

### Buffers and culture materials

Decontamination washes were made fresh on the day of use by adding 500 µl Gibco Penicillin-Streptomycin-Glutamine (100×) and 500 µl of Gibco Amphotericin B (25 ng/ml) to 50 ml sterile phosphate buffered saline (PBS) at room temperature.

Artificial seawater (ASW) was made by adding 35 g Tropic Marin©Classic Sea salt (Wartenberg, Germany) to one litre of Milli-Q^®^ purified water, mixing to complete dissolution, and autoclaving. ASW was stored at room temperature prior to use. Oyster cell culture media was made by mixing 25 ml Leibovitz’s L15 media (this can be replaced with Opti-MEM reduced serum medium if required for experiments) with 25 ml ASW, supplemented with Gibco Penicillin-Streptomycin-Glutamine to 50 µg/ml and Gibco Amphotericin B to 25 ng/ml. Media was passed through a 0.22 µm syringe filter and stored at 4 °C for up to 2 weeks.

### Tissue culture vessel coatings

Plastic vessels for tissue culture were coated with either Matrigel or Poly-d-lysine. Matrigel^®^ matrix 10 mg/ml was thawed overnight on ice and diluted with Opti-MEM reduced serum medium to 2 mg/ml aliquots. Plastic vessels were covered evenly with minimum volume of 2 mg/ml matrigel mix and incubated for at least 60 min at 28 °C.

Poly-d-lysine (1 mg/ml) was thawed and diluted with Opti-MEM reduced serum medium into 0.1 mg/ml aliquots. Plastic vessels were covered evenly with minimum volume of 0.1 mg/ml poly-d-lysine and incubated for at least 60 min at 28 °C prior to use in tissue culture.

### Oyster cell passage

Cell passage was tested with trypsin EDTA (1×), collagenase (1 mg/ml), TrypLE™ (1×) and thorough rinsing with PBS. Each approach was done separately on matrigel coated, poly-d-lysine coated and uncoated plastics. To passage cells for sub-culturing, media was carefully removed and cells were gently rinsed with PBS, which was added to the side of the wells to prevent lifting cells. PBS was removed without disturbing cells. Dissociation reagent (pre-warmed to 37 °C) was added and cultures were incubated at 28 °C for up to 5 min. An equal volume of oyster media was then added to the cell suspension to neutralise the dissociation reagent. Cell suspension was centrifuged at 3,000 g for 5 min and supernatant was removed. Cell pellet was resuspended in oyster media and reseeded.

### Cell imaging

Cells were kept out of light as much as possible during imaging and were removed from incubator for a maximum of 60 min. Still images and videos of tissue contractions were taken using a Zeiss Axiovert 40 inverted fluorescent microscope, Zeiss MRm mono camera, and Zen Software. Cells were imaged inside tissue culture vessels to prevent stress. Live cell imaging was achieved using Zeiss inverted live cell observer microscope in gassed chamber at 28 °C and 5% CO_2_, Zeiss MRm low light camera, and Zen Blue Pro software.

### Quantification of gene expression

Quantitative PCR (qPCR) was used to confirm that cells in culture were active via gene expression. Mantle, adductor muscle and gill from three adult Pacific oysters were dissected into pieces 2 to five mm across and cultured in 12 well plates following the methodology above using Matrigel coated wells. Three separate cultures were established for each animal for each time point (0, 1, 5 and 9 days post establishment), for a total of 9 replicates (3 biological × 3 technical) per tissue per time point. Hearts from 12 animals were cut into four equal pieces. Heart sections were randomly assigned into 9 wells per time point. At appropriate time points, tissue was fixed with Trizol following the manufacturer’s instructions and stored at −80 °C before RNA isolation. Day 0 samples included the tissue explant in Trizol extraction, whereas all other time points did not. After isolation, RNA was DNAse treated using RNAse-Free DNAse (Qiagen, Hilden, Germany) following the manufacturer’s instructions and quantified using Nanodrop. cDNA was synthesised using High-Capacity cDNA (Applied Biosystems™, California, United States) reverse transcription kit following manufactures instructions. Ribosomal protein L7 was selected as gene of choice, based on validation and primers from Du et al. (2013) (5′-TCCCAAGCCAAGGAAGGTTATGC-3′5′-CAAAGCGTCCAAGGTGTTTCTCAA-3 ′). qPCR was performed using RotorGene-3000 in reaction volumes of 20 µl. Each reaction contained 2 µl cDNA with 18 µl of FastStart Universal SYBR Green Master (Rox) with both RL7 primers, cDNA and water to manufactures instructions. PCR program consisted of: 95 °C for 10 min; followed by 40 cycles of 95 °C for 25 s, 60 °C for 15 s and 72 °C for 20 s; 94 °C for 20 s; 65 °C ramp to 94 °C by 1 °C after 60 s then every 5 s subsequently. Results were recorded as raw qt (quantification threshold) values.

### DNA Barcoding of cell cultures

To confirm that cultures originated from Pacific oyster, DNA was extracted from cultures using Trizol following the manufactures instructions. Barcoding was performed using custom primers CO1-F 5′-CATGCAATTTCCTCGATTAAATGCA-3′ and CO1-R 5′-AGACGTAGTGAAAATGACCAGTTACA-3′ based on *C. gigas* cytochrome oxidase-1 (CO1) gene sequence from NCBI GenBank. PCR was performed in 20 µl reaction volumes with 10 µl BioMix Red, 0.5 µl of each primer (10 µM), 1 µl DNA and 8 µl DNAse free water. Thermal cycling comprised of 95 °C for four minutes; 35 cycles of 95 °C for seconds, 56 °C for 30 s, 72 °C for 30 s; 72 °C for 10 min. PCR products were run on 1% agarose gel and imaged using Syngene™ G:BOX. Positive and negative controls for CO1 were used for each run.

## Results

**Figure 1:**
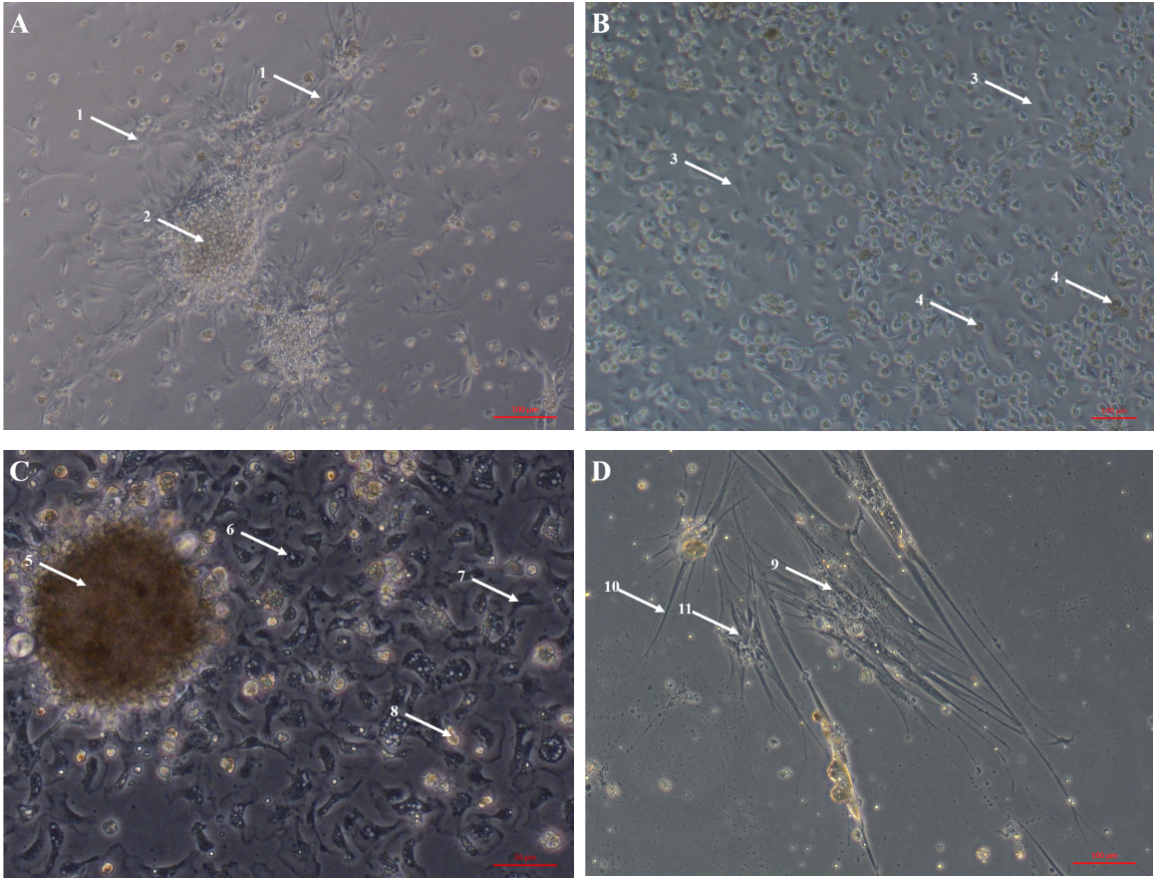
Primary cell cultures established from Pacific oyster heart explants. Bright field images of primary cell cultures established from oyster heart explants. (A) Heart primary culture clearly showing fibroblast-like cells (1) dissociating from aggregations of rounded cells (2) and adhering to culture plastics. (B) High confluency heart primary culture 28 days after establishment fibroblast-like cells (3) and rounded cells (4) (C) Heart primary culture 12 days post establishment from tissue fragment (5) with confluent granulocytes (6), agranulocytes (7) and rounded cells (8) (D) Cardiomyocyte like cells in culture. Cells display long extending protrusions (10) and complex internal structures (11).

**Figure 2:**
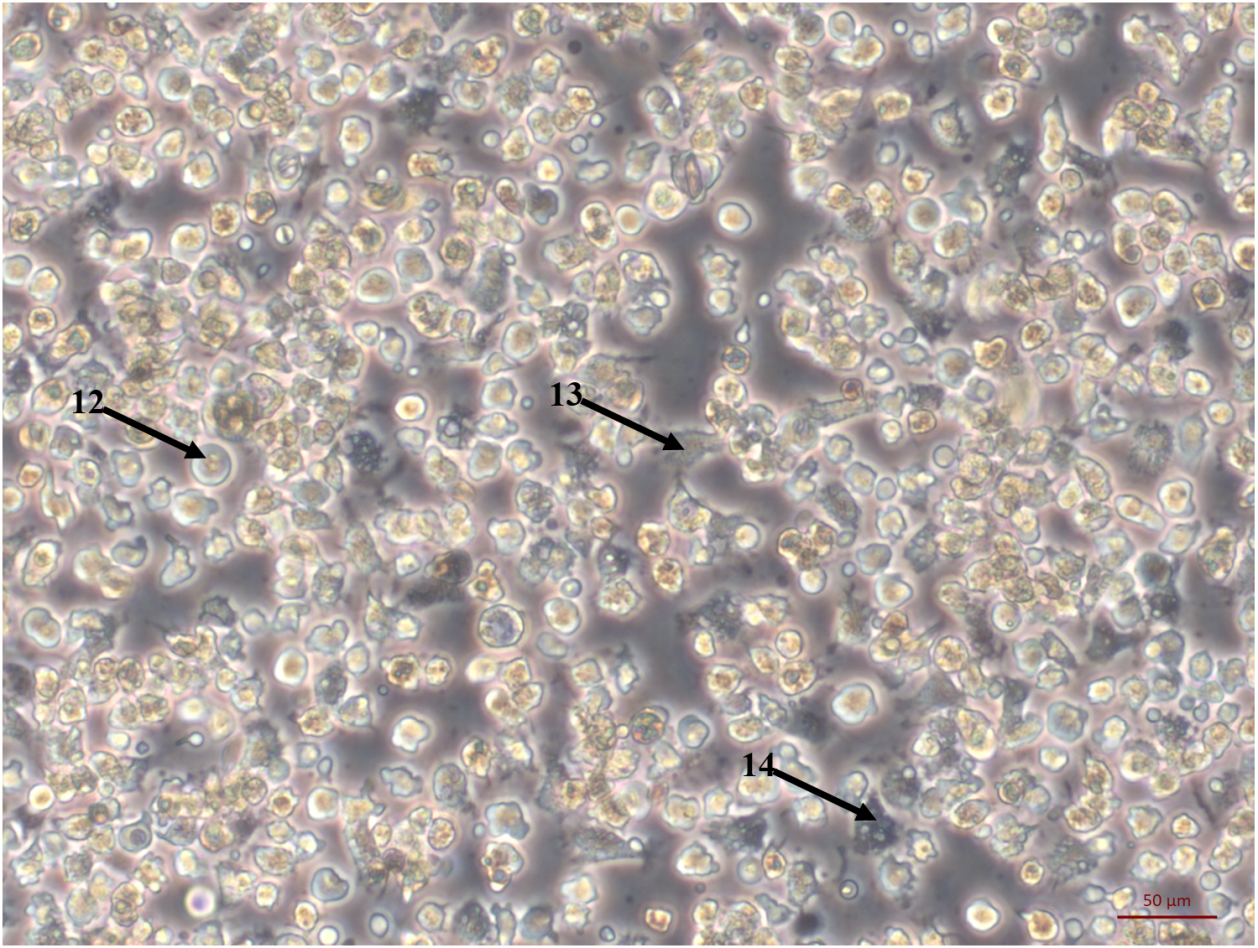
Primary cell culture of Pacific oyster hemocytes. Primary cell culture of Pacific oyster hemocytes established from hemolymph showing rounded cells (12), agranulocytes (13) and granulocytes (14).

Primary cell cultures established from heart explants showed the greatest variety of cell morphologies, including cells resembling piscine fibroblast cells originating from a tissue fragment that are adherent to the tissue culture plates (Fig. 1A) (Gao et al., 2019). Rounded cells are also visible away from the tissue fragment which resemble cells cultured from mussel (*Mytilus edulis)* mantle (Daugavet & Blinova, 2015). Some of these cells may not be fully adhered to the tissue culture plates, similar to hemocytes in culture (Fig. 2). Over time, tissue fragments broke down and cultures became more confluent. Multiple cell morphologies were present after 28 days, similar to the earlier cultures (Fig. 1B). Hemocyte-like cells were present in many of the cell cultures established from oyster hearts (Fig. 1C). Hemocytes have multiple morphologies, but are broadly classified into granulocytes, semigranulocytes and agranulocytes, with the latter being more prevalent in older cultures (Jiang et al., 2016) (Fig. 2). Some more established heart cultures also had cells that were elongated, similar to dedifferentiated, proliferating cardiomyocytes from zebra fish heart culture (Fig. 1D) (Sander et al., 2013). Cultures established from hearts using this large explant method were maintained with a healthy appearance for up to 6 weeks (i.e., not just rounded cells).

**Figure 3:**
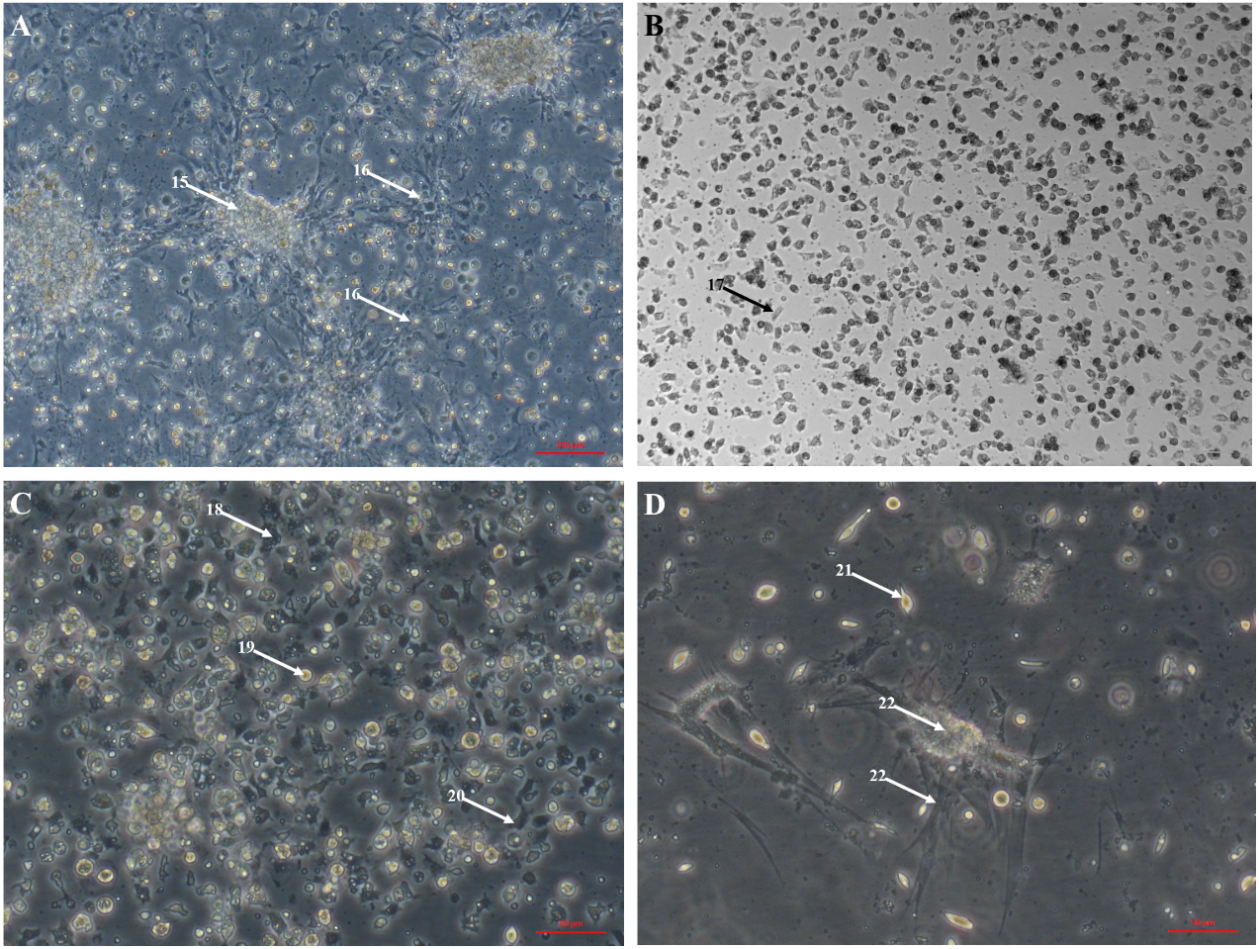
Primary cell cultures established from multiple Pacific oyster tissues. Bright field images of primary cell cultures established from multiple different tissues. (A) Mantle primary culture with aggregations of cells (15) and networks of fibroblast-like cells dissociating and adhering to culture plastics (16). B) Gonad primary culture with fibroblast-like cells visible (17). C) Gill primary culture with granulocytes (18), rounded cells (18) and fibroblast-like cells (20) all visible. D) Muscle primary culture with spindle shaped cells (21), as well as larger cell mass (22) with elongated protrusions adhering to culture plastics (23).

The large explant method was also successful for culturing cells from other tissues dissected from the oyster. Mantle primary cell culture (Fig. 3A) showed similar morphology to heart fibroblast-like cell culture (Fig. 1A). Previous studies of molluscan mantle primary cell culture have not reported any fibroblast-like cells. Rounded cells were also visible (Fig. 3A), which have been reported previously in mussel cell culture (Daugavet & Blinova, 2015). Primary cell cultures established from gonad tissue were established for the first time in *C. gigas.* Unlike heart and mantle cultures the cells were isolated, with granulocytes, rounded and fibroblast-like cells all visible (Fig. 3B). Gonad cultures had high frequency of contamination and so were not maintained for longer than 7 days. Primary cell cultures established from gill tissue had similar appearance to both hemocyte and gonad cultures with many granulocytes, rounded and fibroblast-like cells all visible (Fig. 3C). Unlike hemocyte and gonad cultures, gill cultures were maintained for up to 10 weeks, after which time only rounded cells were visible. Primary cell cultures established from adductor muscle tissue were also established for the first time in *C. gigas.* Both smooth and striated muscle tissue were used to establish cultures, which show rounded and spindle-shaped cells, as well as larger muscle-like cells (Fig. 3D).

Hearts dissected from adult oysters were visibly beating up to eight weeks after transfer to culture conditions (see Video S1). Papillae on the edges of mantle explants dissected from adult oysters were visibly motile up to two weeks after transfer to culture conditions. Cilia around the edges of gill tissue fragment exhibited characteristic wave-like movement after 4 weeks in culture conditions. This novel discovery may provide a new model for studying shellfish biology. No visible tissue movements were observed in adductor muscle, digestive gland or gonad explants.

Both poly-d-lysine and matrigel coating of plastic culture vessels seemed to alter the assemblages of cells that were visible in primary cell cultures established from mantle, gill and adductor muscle, allowing for more frequent culture of fibroblasts, spindle shaped cells and larger cells, which were rarely visible on uncoated plastics. Pre-coating plates did not have a clear visible effect on heart cultures, which had adherent cells on uncoated TC plastic.

**Figure 4:**
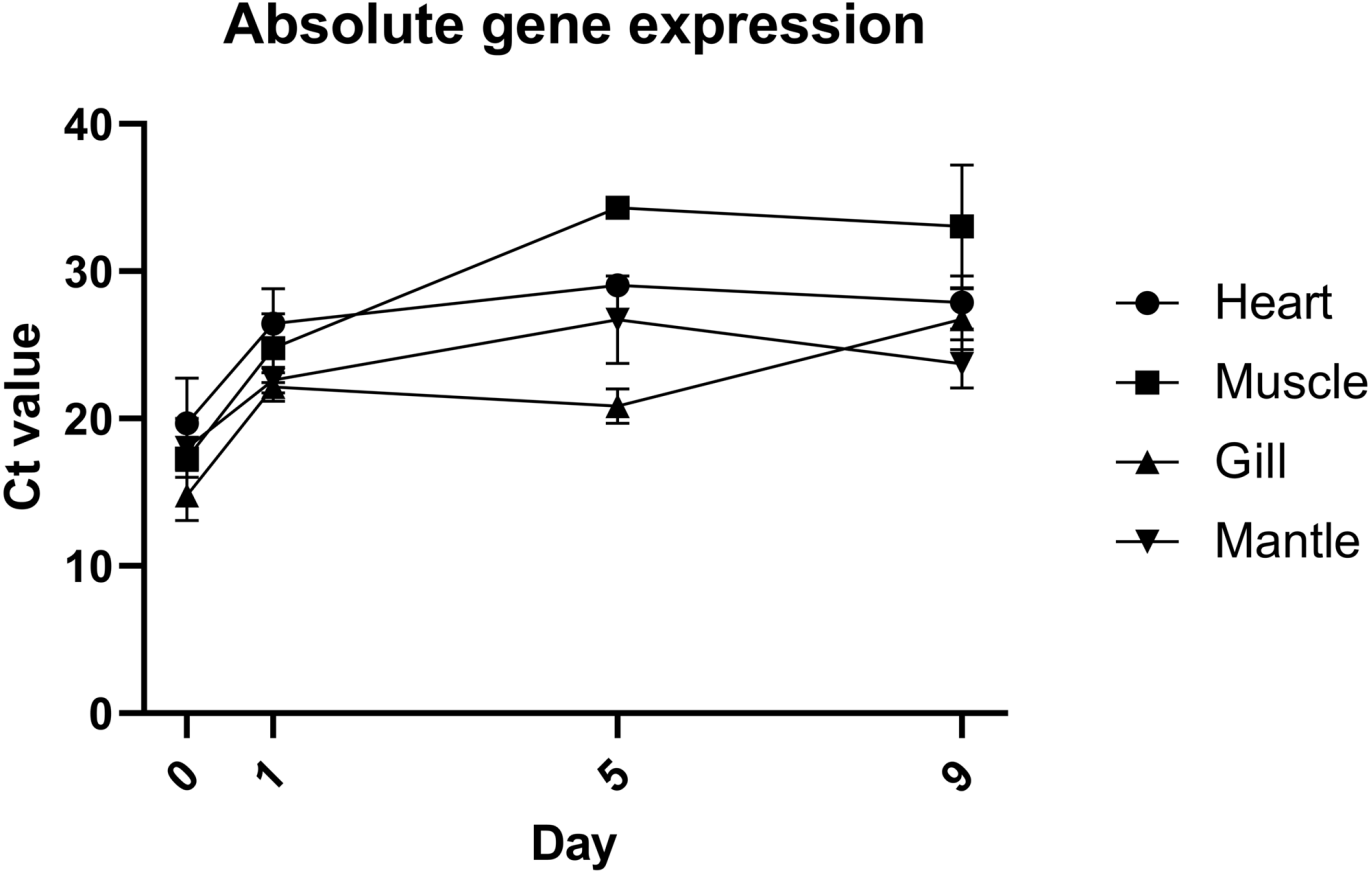
Absolute expression of a housekeeping gene in oyster primary cell culutre from multiple tissues. Gene expression of primary cell cultures established from heart, adductor muscle, gill and mantle tissues dissected from Pacific oyster over time. Tissue explants were included in total RNA extraction for 0 days post establishment, but not for subsequent time points.

Quantification of gene expression suggests that there is no biologically significant difference in gene expression between days 1 and 9, which supports the evidence from morphological assessment of cultures over time (Fig. 4). Additionally, this study is the first to visualise primary cells of any mollusc species using live cell imaging (see Video S2). Cells appear to be highly active, further suggesting that cultures established with this method are metabolically active and that the media is suitable. Previous molluscan primary cell cultures have used cell adherence alone as evidence for metabolic activity (Quinn et al., 2009; Daugavet & Blinova, 2015; Le Pennec & Le Pennec, 2001). DNA barcoding results showed that cultures were indeed from *C. gigas.*

## Discussion

The large explant method described here allowed repeated establishment of highly confluent cell cultures with a healthy appearance from multiple different tissues and demonstrate an advance in the state-of-the-art of molluscan primary cell culture. The two key differences between the large explant method and previous methods to establish marine molluscan cell cultures are the use of strong universal biocides (i.e., bleach) and no tissue dissociation stage.

The results here suggest that the large explant method developed has benefits over previously reported culture methods using tissue dissociation (Boulo et al., 1996; Boulo et al., 2000; Chen & Wang, 1999; Domart-Coulon et al., 1994; Le Deuff, Lipart & Renault, 1994; Yoshino, Bickham & Bayne, 2013) or small tissue explant (Daugavet & Blinova, 2015), providing a greater range of methods available for investigating molluscan primary cell culture. This is primarily due to the presence of novel cell morphologies that have not been reported previously in molluscs, and reduced frequency of contamination from bacteria and other marine organisms. Using the large explant method does not require use of dissociation reagents, which can negatively impact cells. The lower prevalence of contamination in this study may also be due in part to the use of large tissue explants, which allows for more rigorous decontamination as some cell-death does not prevent establishment of the culture. It is difficult to quantify the levels of contamination between studies, as cultures with evidence of contamination are generally discarded immediately to prevent cross contamination. However, within this study, cultures established using the dissociation method (see Text S1) (Le Deuff, Lipart & Renault, 1994), had a much higher frequency of contamination compared to cultures established using the large explant method. Most of the heart cultures that were initially established with antibiotic supplemented media, then later in antibiotic free media, remained contamination free throughout. The method described here is simpler than previous molluscan cell culture methods, as it does not require use of serum or other additives (Domart-Coulon et al., 1994), ability to produce oyster larvae (Boulo et al., 2000) or the availability of natural seawater (Le Deuff, Lipart & Renault, 1994).

There is scope for further optimisation of the culture conditions for molluscan primary cells. As discussed, the complexity of the marine environment variability of conditions within the pallial cavity makes identifying optimal culture conditions difficult. Furthermore, optimal conditions may vary for different species or even different populations due to habitat variation and local adaptation. All the passage reagents tested here were similarly effective, and have significant scope for improvement in terms of cell recovery and efficacy. After passage, fewer cell morphologies were visible in primary cultures. It may be that certain cells dissociated from the explant cannot be passaged as they are differentiated non-replicating cells; similar to cultured hemocytes (Terahara, Takahashi & Mori, 2003; Terahara, Takahashi & Mori, 2005).

This explant culture system may be applicable to studying many aspects of oyster biology, but in particular in may be advantageous for Ostreid herpes virus (OsHV) replication, the most economically important Pacific oyster pathogen, as it represents a higher level of organisation than culture of a single cell type. Hemocyte culture has been used to increase quantity of viral DNA, but the crucial step of viral isolation and reinfection has not been reported (Morga et al., 2017). Evidence suggests that hemocytes are involved in the OsHV replication cycle, but other cell types may also be necessary. If this is the case, then using the whole organ system which has been developed here may worthy of further investigation into OsHV virology. A similar system has recently been developed from grass carp, also using an explant method, which has been suggested as a useful tool for toxicological, physiological and medical studies (Rastgar et al., 2019).

Knowledge of molluscan cell morphology is limited, with previous cultures displaying mostly rounded cells and hemocytes. Comparison with mammalian cells suggests myoblasts have been cultured from a mollusc for the first time here (Tesseraux, Gülden & Wassermann, 1987). Cells resembling mammalian cardiomyocytes were also observed. Interestingly they did not look identical to cells previously identified as cardiomyocytes from oyster cell culture (Kimes & Brandt, 1976; Le Deuff, Lipart & Renault, 1994). Classifying different cells is currently reliant on limited morphological assessment; comparing bright field or phase contrast images of cultures. Conclusions are therefore based on the appearance of cultures, the source of cells and their behaviour over time e.g., how long cells remain adherent. Improved understanding and applicability of methods to characterise molluscan cells would be beneficial to distinguish between the different cell morphologies that have been cultured both here and in previous studies. For example, characterisation of mammalian cells can be achieved using staining, immunofluorescence and flow cytometry (Alvi et al., 2002). Flow cytometry has been used to characterise *C. gigas* hemocytes, demonstrating this approach can be applied to molluscan cells (Jiang et al., 2016). In experimentally challenging mammalian cells clusters of differentiation and qPCR can be used to characterise a population of cells (Cortes et al., 2018). These approaches have not been developed in molluscan culture, making it difficult to determine what cell types have been cultured beyond morphological characterisation.

Lack of basic culture methods for establishing and characterising oyster primary cultures is a key limitation. An effective quantitative non-lethal cell proliferation assay would be beneficial for determining the effect of different culture methods. Passage and cell counts are currently ineffective and damaging to cell cultures, which is a major bottleneck to attempting the immortalisation approach that was successful for the Bge cell line. Further, the specific culture conditions required for molluscan cells have not been optimised. The presence of dissected heart and mantle contractions weeks after transfer to culture conditions suggests that the final media refined here is an improvement on previously used media.

Establishment of an immortalised cell line remains a key goal in shellfish research. Due to difficulty of working with primary cell cultures from oysters, it may be necessary to induce immortalisation artificially rather than to encourage spontaneous immortalisation. This approach would still require an effective and reliable method for establishing primary cultures as a starting point for the immortalisation. There are currently no reports of attempts to artificially induce immortalisation in molluscan cells, however new developments in cell immortalisation in recent years may offer new approaches. The large explant method facilitates this, and also provides a greater range of tissues and cell types as targets for immortalisation compared to other methods of culture establishment. One potential method to do this may be to use the CRISPR/Cas9 system to knockout genes involved in the cell cycle e.g., telomerase (Zhu et al., 2007). An alternative strategy would be to mirror techniques effective in insect cell cultures, by using mutagens to establish immortal lines, which may also be applicable to molluscan cells (Li et al., 2012). Cell cultures can also be used to test transgenesis and editing systems without the need to maintain an experimental breeding oyster population. Interest in transgenesis and genome editing in molluscs has increased in recent years due to improvements in technologies (e.g., CRISPR/Cas9), and the potential for these technologies to address production barriers in aquaculture production (Gratacap et al., 2019).

## Conclusions

The large explant method developed here allows the repeated establishment of primary cell cultures from multiple Pacific oyster tissues without alteration. These cultures have novel cell morphologies and greater evidence of metabolic activity than previous culture methods. Additionally, the discovery of whole tissues contracting for weeks after removal from the donor organism presents opportunities for a new paradigm in the challenging field of molluscan cell and tissue culture. The ability to regularly produce axenic primary cultures from multiple tissues offers an increased versatility as well as an increased likelihood of generating an immortalised cell line, and further facilitates the use of marine invertebrate tissue cultures as an experimental model.

##  Supplemental Information

10.7717/peerj.9180/supp-1 Text S1 Supplementary methods for dissociation of oyster and changing culture conditions that were not used to generate any of the figures, but are relevant and interesting experiments that have not been reported previously

10.7717/peerj.9180/supp-2 Video S1 Dissected oyster heart contractions Heart removed from an oyster clearing contracting rhythmically 6 weeks after dissection. Heart is approximately 3mm across.

10.7717/peerj.9180/supp-3 Video S2 Live cell imaging of oyster heart cells Live cell imaging of Pacific oyster heart culture 1 day post establishment, showing fibroblast-like cells and hemocytes adhering and moving across tissue culture plastic. Scale bar = 900 *μ*m. Real time is displayed top left.

10.7717/peerj.9180/supp-4 Data S1 CO1 barcoding sequences from oyster cell cultures

10.7717/peerj.9180/supp-5 Data S2 Raw data from qPCR of oyster cell cultures—used for Fig. 4

10.7717/peerj.9180/supp-6 Figure S1 Pacific oyster anatomy diagram Anatomy of the pacific oyster with labels for all major tissues visible. Digestive gland (not visible) is found enveloped by gonadal tissue. Top (right) layer of mantle has been removed.

10.7717/peerj.9180/supp-7 Figure S2 Anatomy of oyster (Provided to give an original)

10.7717/peerj.9180/supp-8 Figure S3 Multiple cell morphologies from oyster heart primary culture. Part 1 of 4—fibroblast-like cells Heart primary culture clearly showing fibroblast-like cells dissociating from aggregations of rounded cells and adhering to culture plastics.

10.7717/peerj.9180/supp-9 Figure S4 Multiple cell morphologies from oyster heart primary culture. Part 2 of 4—highly confluent cells High confluency heart primary culture 28 days after establishment with round, spindle shaped and epithelial-like cells all visible.

10.7717/peerj.9180/supp-10 Figure S5 Multiple cell morphologies from oyster heart primary culture. Part 3 of 4—hemocytes surrounding explant Heart primary culture 12 days post establishment with confluent granulocytes, with round and epithelial-like cells also visible.

10.7717/peerj.9180/supp-11 Figure S6 Multiple cell morphologies from oyster heart primary culture. Part 4 of 4—large elongated cells Large elongated cardiomyocyte like cells. Hemocytes, round, spindle shaped and epithelial-like cells are also visible.

10.7717/peerj.9180/supp-12 Figure S7 Oyster primary cell cultures established from large explants of multiple tissues. Part 1 of 4—mantle primary culture Mantle primary culture showing epithelial-like cells dissociating from aggregations of rounded cells and adhering to culture plastics, similar to heart cultures.

10.7717/peerj.9180/supp-13 Figure S8 Oyster primary cell cultures established from large explants of multiple tissues. Part 2 of 4—gonad primary culture Gonad primary culture with round and fibroblast-like cells visible, 5 days post explant.

10.7717/peerj.9180/supp-14 Figure S9 Oyster primary cell cultures established from large explants of multiple tissues. Part 3 of 4—gill primary culture Gill primary culture with hemocytes, round and fibroblast-like cells.

10.7717/peerj.9180/supp-15 Figure S10 Oyster primary cell cultures established from large explants of multiple tissues. Part 4 of 4—adductor muscle primary culture Muscle primary culture with round, spindle shaped and larger muscle-like cells adhering to the culture plastics.

## Additional Information and Declarations

### DNA Deposition

The following information was supplied regarding the deposition of DNA sequences: The sequences are available as a Supplemental File.

### Data Availability

The following information was supplied regarding data availability: All qPCR data and sequences are available in the Supplemental Files.
