# Supplementary material for: Developments in marine invertebrate primary culture reveal novel cell morphologies in the model bivalve Crassostrea gigas
Source: PeerJ. 2020 Jun 1;8:e9180. doi: 10.7717/peerj.9180 (PMC7271890; doi:10.7717/peerj.9180)
Supplement: Text S1 [file peerj-08-9180-s001.docx]

**Supplementary text**

**Dissociation of whole oyster**

Juvenile pacific oysters (30-70 mm across) obtained from a UK oyster farm were placed into a decontamination bath of 1:1666 bleach in artificial sea water (ASW) for one hour before being transferred to laminar flow. Tissues were dissected using sterile forceps and incubated for 60 minutes in a solution of trypsin diluted with a solution of 50:50 Leibovitz’s L15 and ASW. Tissue was vortexed regularly and passed through a metal sieve to remove debris. Homogenate was centrifuged at 3000 g for 5 minutes. Supernatant was discarded and cell pellet was suspended in 50:50 L15 and ASW supplemented with Gibco Penicillin-Streptomycin-Glutamine to 50 µg/mL and Gibco Amphotericin B to 25 ng/mL. Cell solution was cultured in T25 flasks at room temperature in a dark box. Flasks with and without poly-d-lysine pre-coating were used. Cell cultures were examined regularly to identify the most suitable time for passaging cells based on the method used by Hansen (1976). Half of the culture media was removed and replaced with fresh media every 3-4 days. Whole oyster dissociated protocol was superseded by explant method due to frequent contamination.

Hemolymph can be stored at 4ºC.

**Changing culture conditions**

Using the explant method above, a range of different culture conditions were tested and examined with light microscopy as a measure of quality. Tests were conducted in 24 well plates with PBS in all outside wells to maintain a constant environment across the different treatments within the plate. For all coating solutions and growth factors, concentrations and preparation methods followed the manufacturer’s instructions. Cultures were examined by light microscopy at regular intervals to gauge health and proliferation of cultures. No clear effect was detected from the addition of serum (5/10/20 % foetal bovine serum), *C. gigas* hemolymph or gonad extract except to increase frequency of contamination. Addition of human epithelial and fibroblastic growth factors (5 ng/ml) had no clear effect. Incubating cultures at 22 ºC did not improve the quality or longevity of cultures and resulted in slower development. Pre coating wells with gelatine and collagen did not have a clear effect on cultures.
