# Supplementary material for: Developments in marine invertebrate primary culture reveal novel cell morphologies in the model bivalve Crassostrea gigas
Source: PeerJ. 2020 Jun 1;8:e9180. doi: 10.7717/peerj.9180 (PMC7271890; doi:10.7717/peerj.9180)
Supplement: Figure S1 — Anatomy of the pacific oyster with labels for all major tissues visible. Digestive gland (not visible) is found enveloped by gonadal tissue. Top (right) layer of mantle has been removed. [file peerj-08-9180-s006.docx]

Supplementary figure 1) Anatomy of the pacific oyster with labels for all major tissues visible. Digestive gland (not visible) is found enveloped by gonadal tissue. Top (right) layer of mantle has been removed.

Heart

Gonad

Adductor striated muscle

Adductor smooth muscle

Gill

Mantle


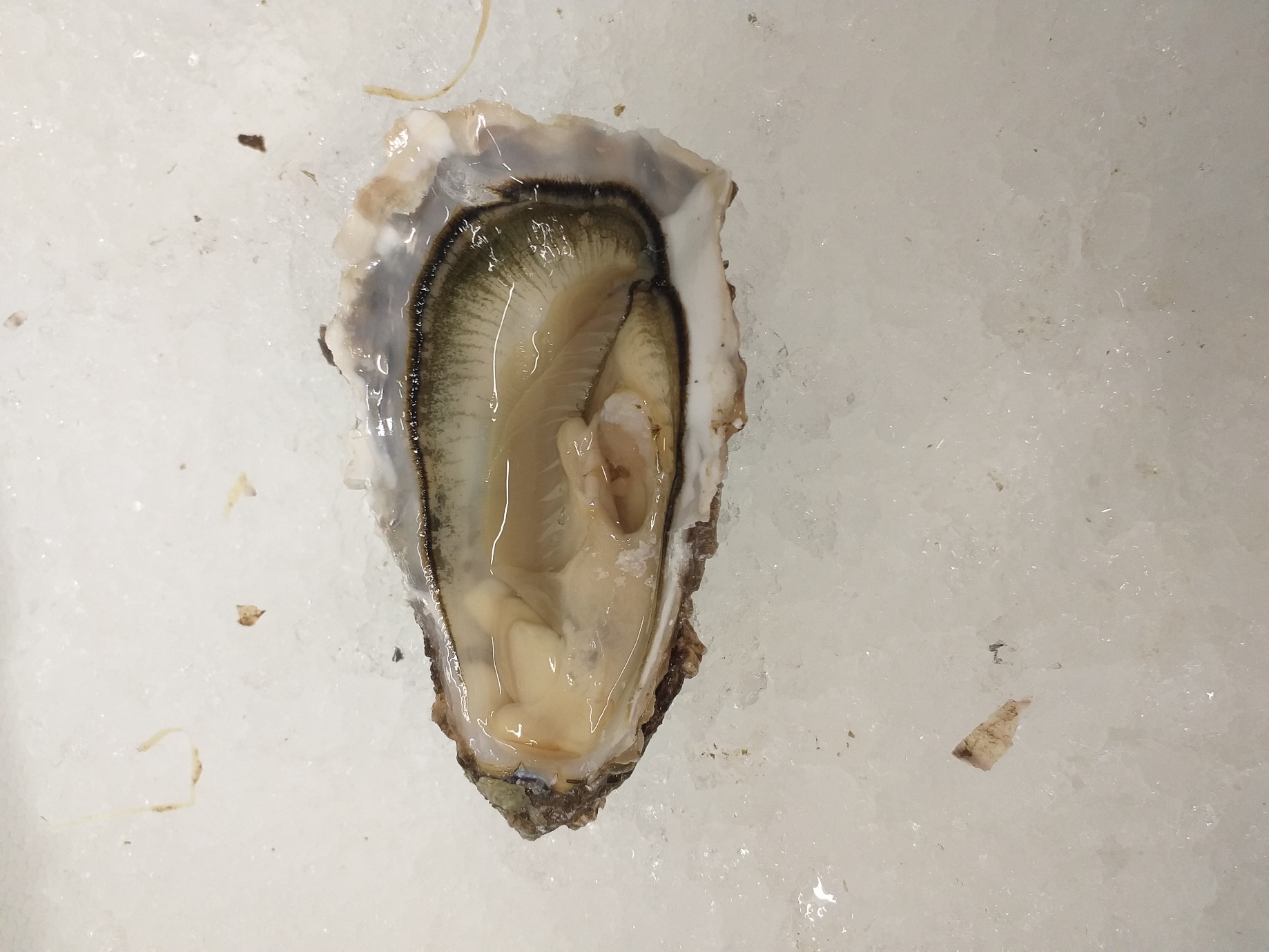


R Potts
