# Supplementary figures and images for: Developments in marine invertebrate primary culture reveal novel cell morphologies in the model bivalve Crassostrea gigas
Source: PeerJ. 2020 Jun 1;8:e9180. doi: 10.7717/peerj.9180 (PMC7271890; doi:10.7717/peerj.9180)

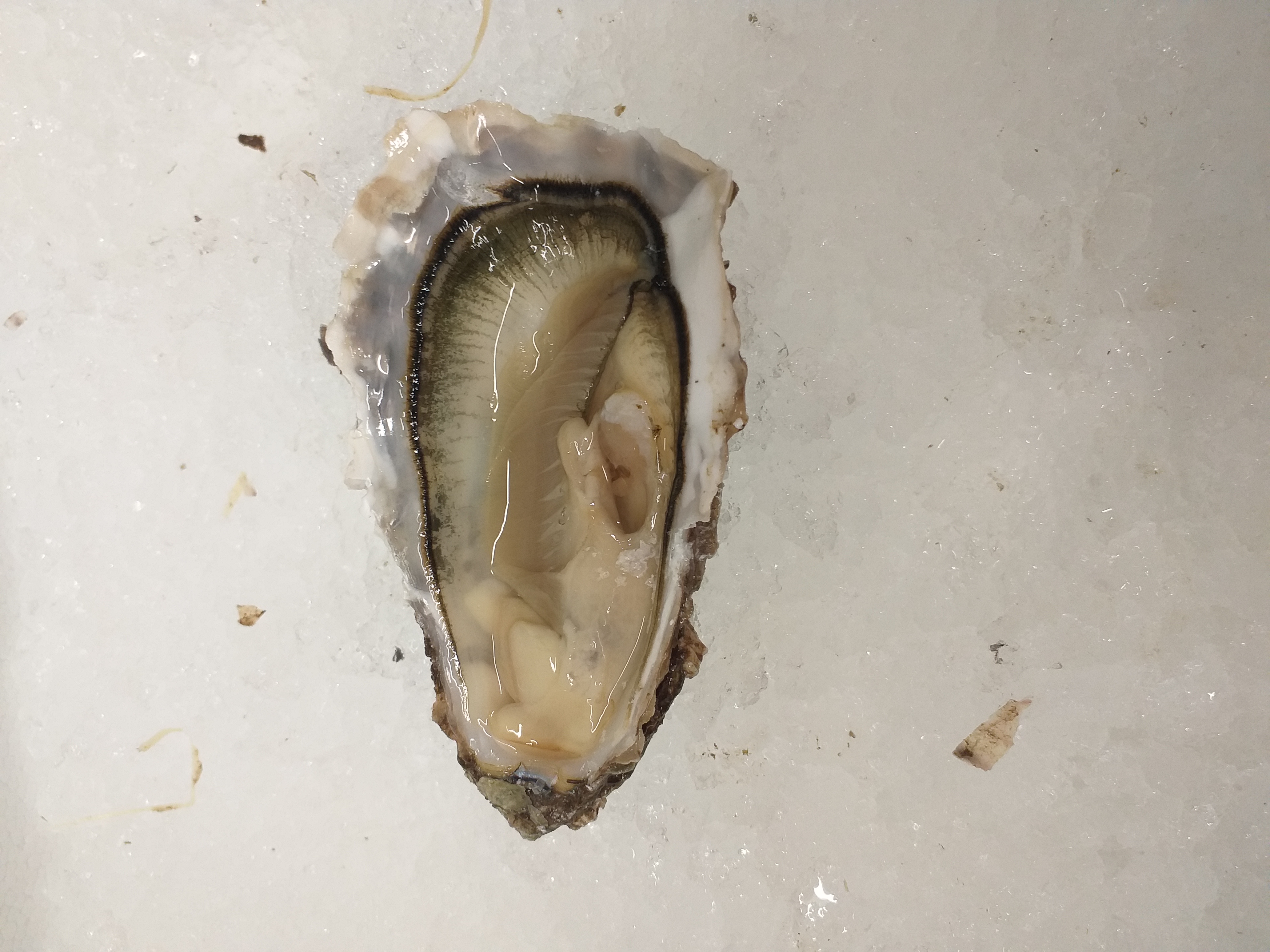

Supplement: Figure S2 — (Provided to give an original) [file peerj-08-9180-s007.jpg]

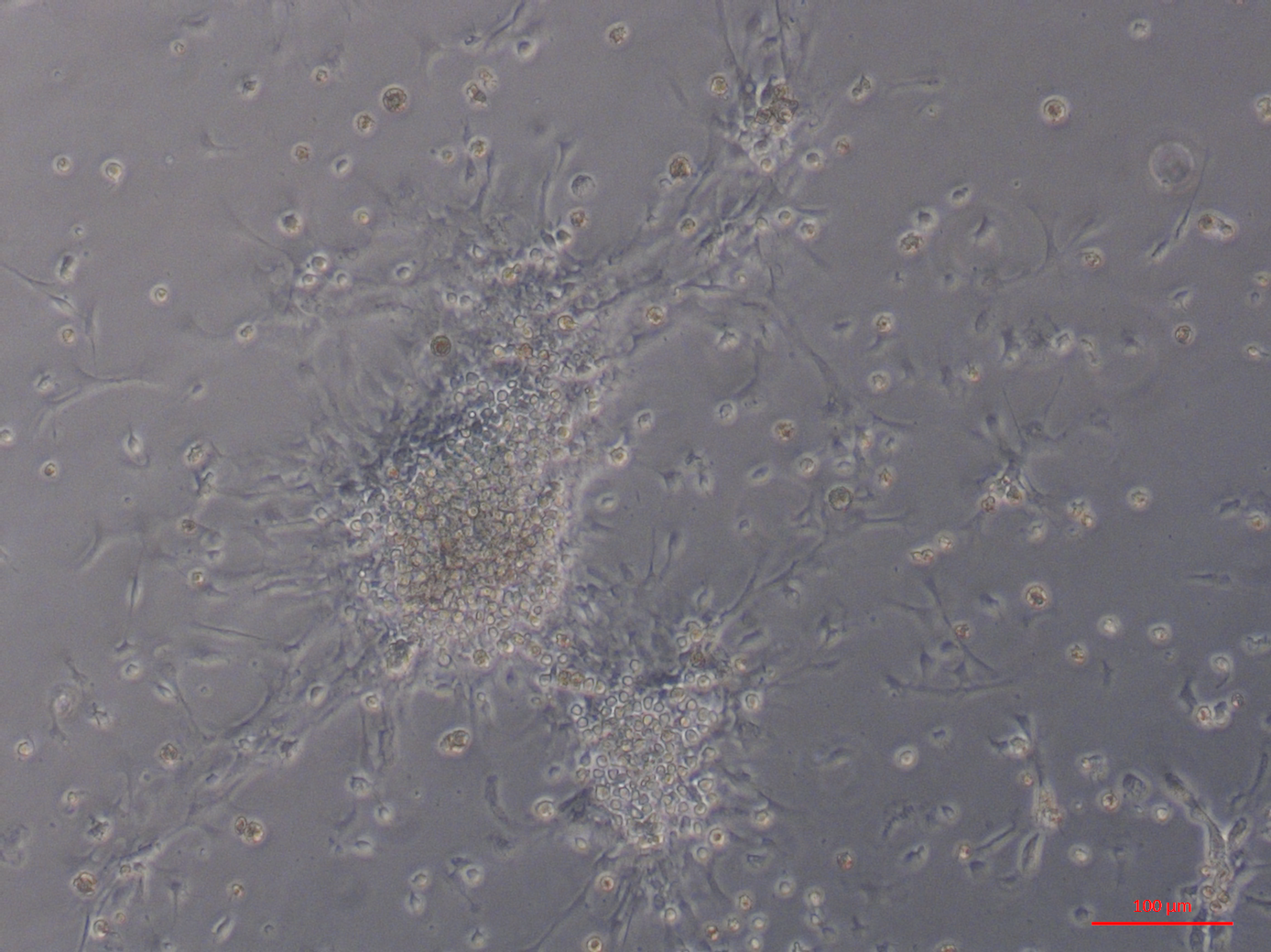

Supplement: Figure S3 — Heart primary culture clearly showing fibroblast-like cells dissociating from aggregations of rounded cells and adhering to culture plastics. [file peerj-08-9180-s008.png]

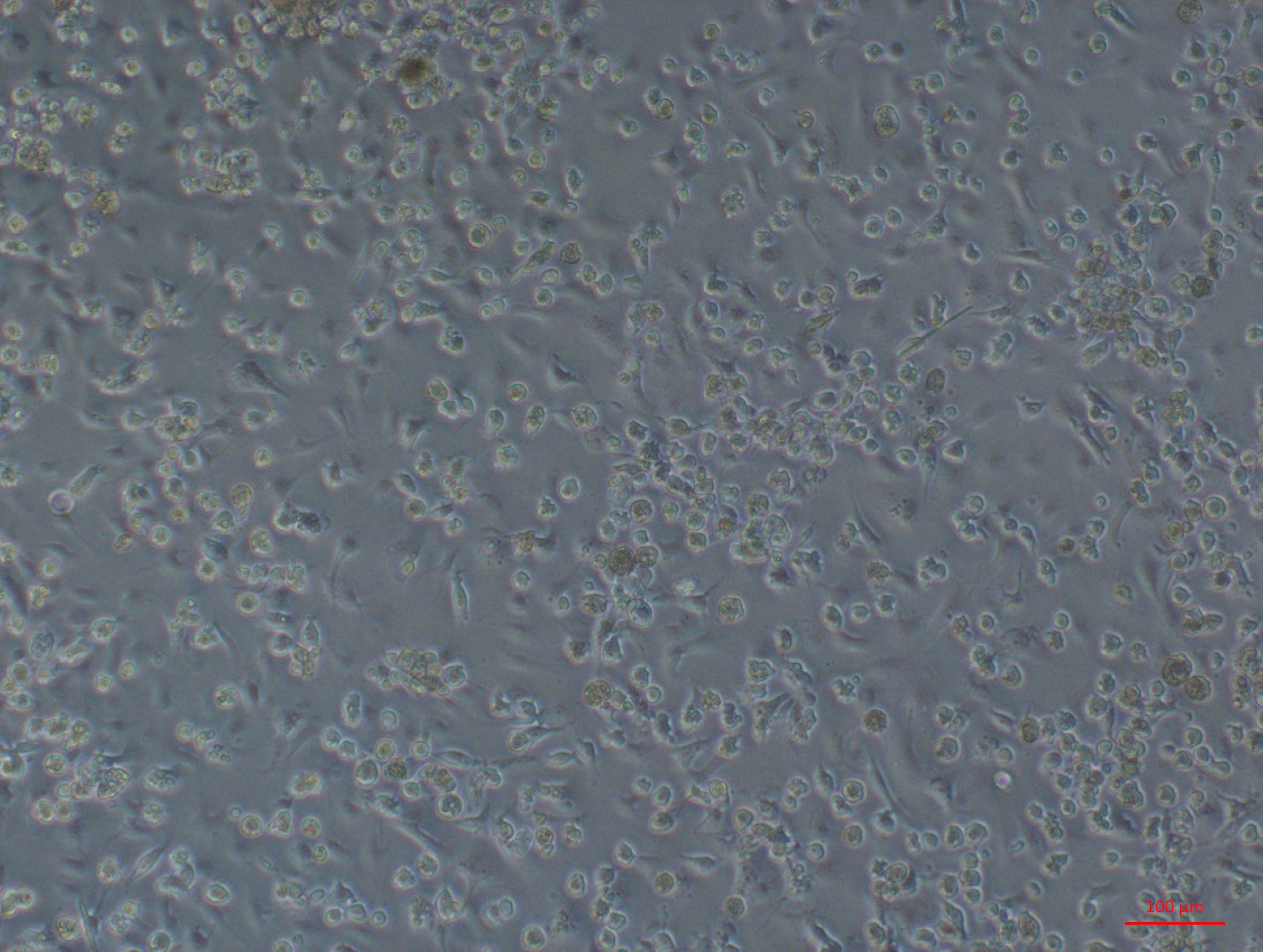

Supplement: Figure S4 — High confluency heart primary culture 28 days after establishment with round, spindle shaped and epithelial-like cells all visible. [file peerj-08-9180-s009.png]

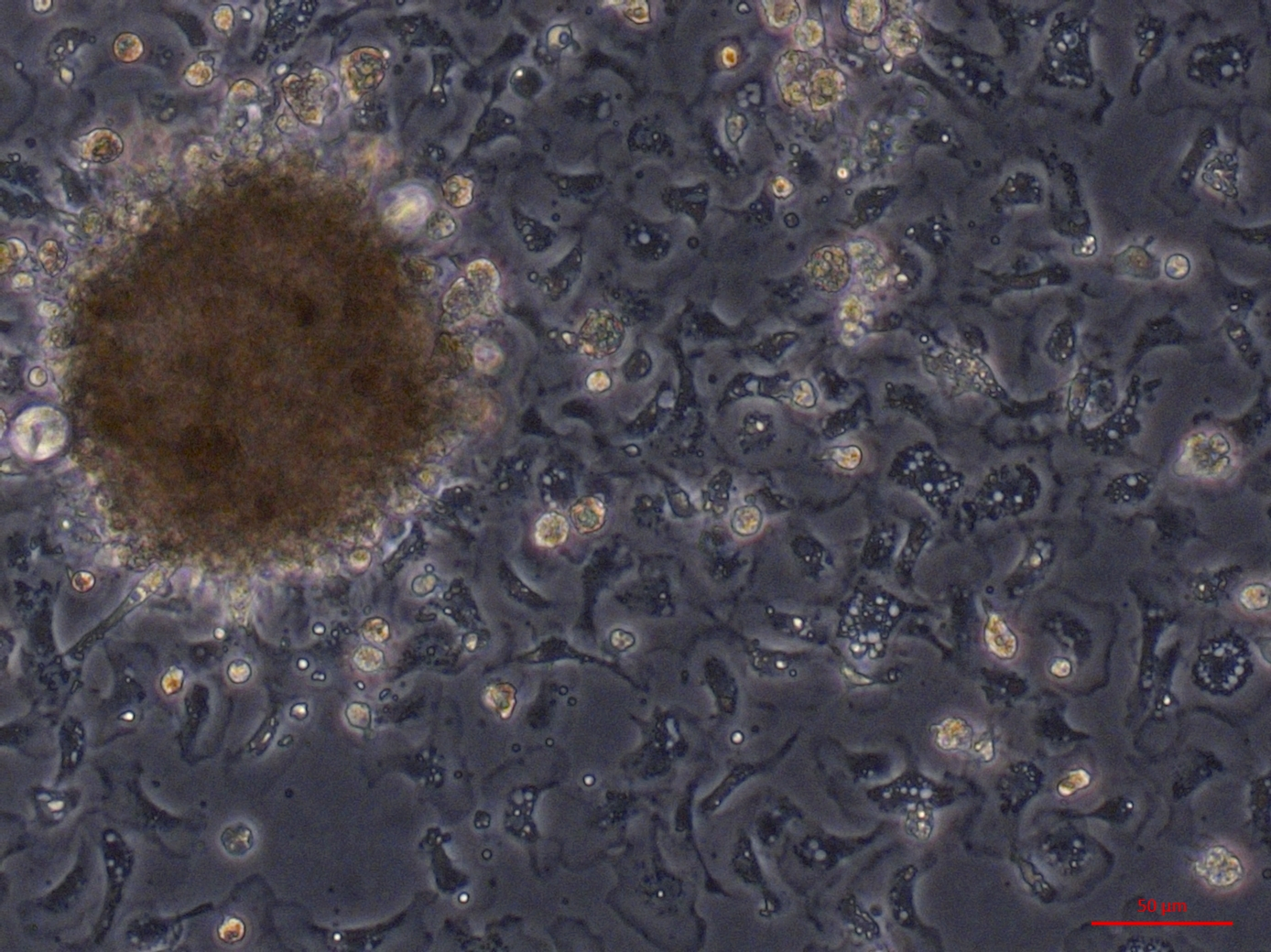

Supplement: Figure S5 — Heart primary culture 12 days post establishment with confluent granulocytes, with round and epithelial-like cells also visible. [file peerj-08-9180-s010.png]

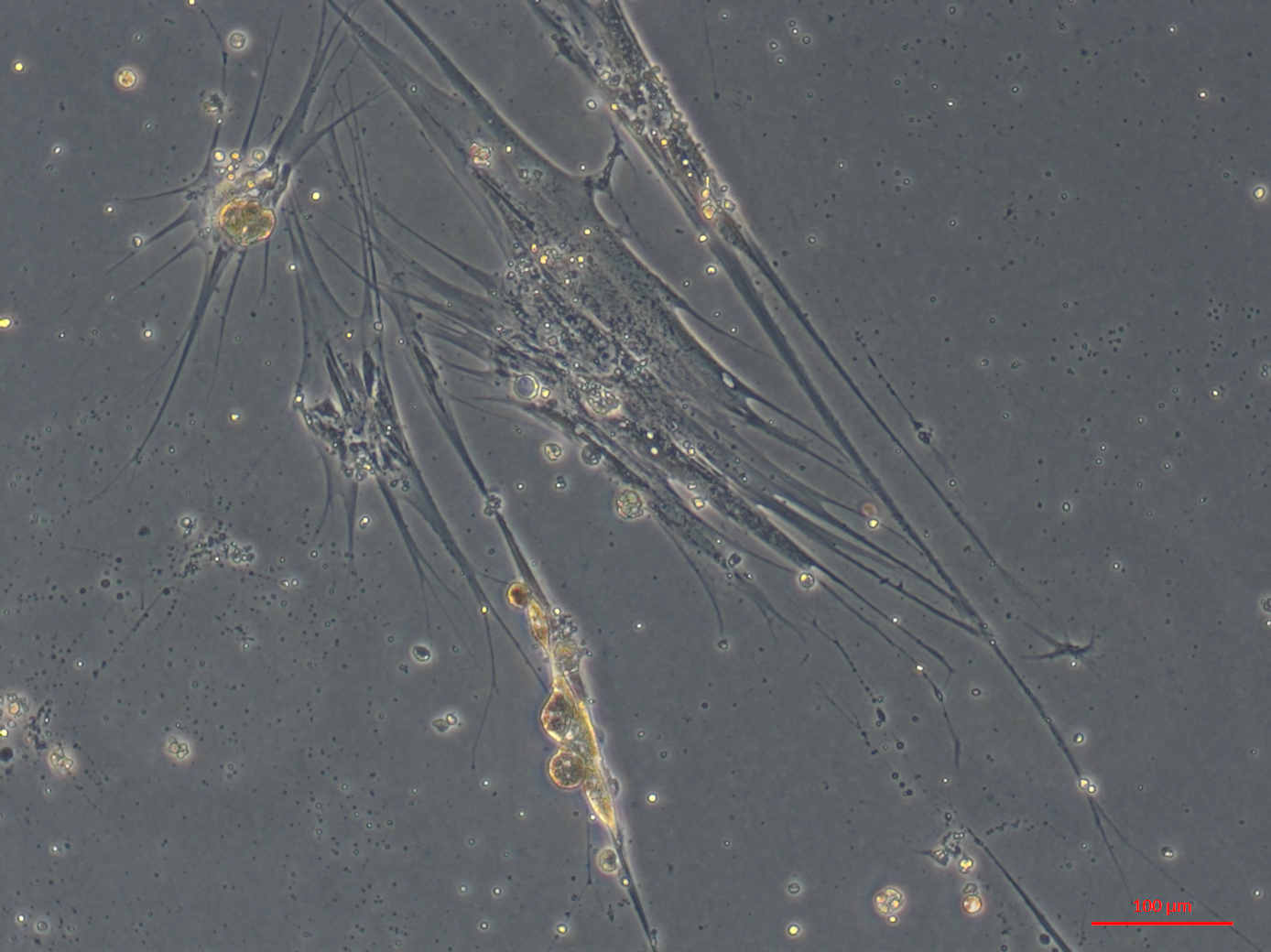

Supplement: Figure S6 — Large elongated cardiomyocyte like cells. Hemocytes, round, spindle shaped and epithelial-like cells are also visible. [file peerj-08-9180-s011.png]

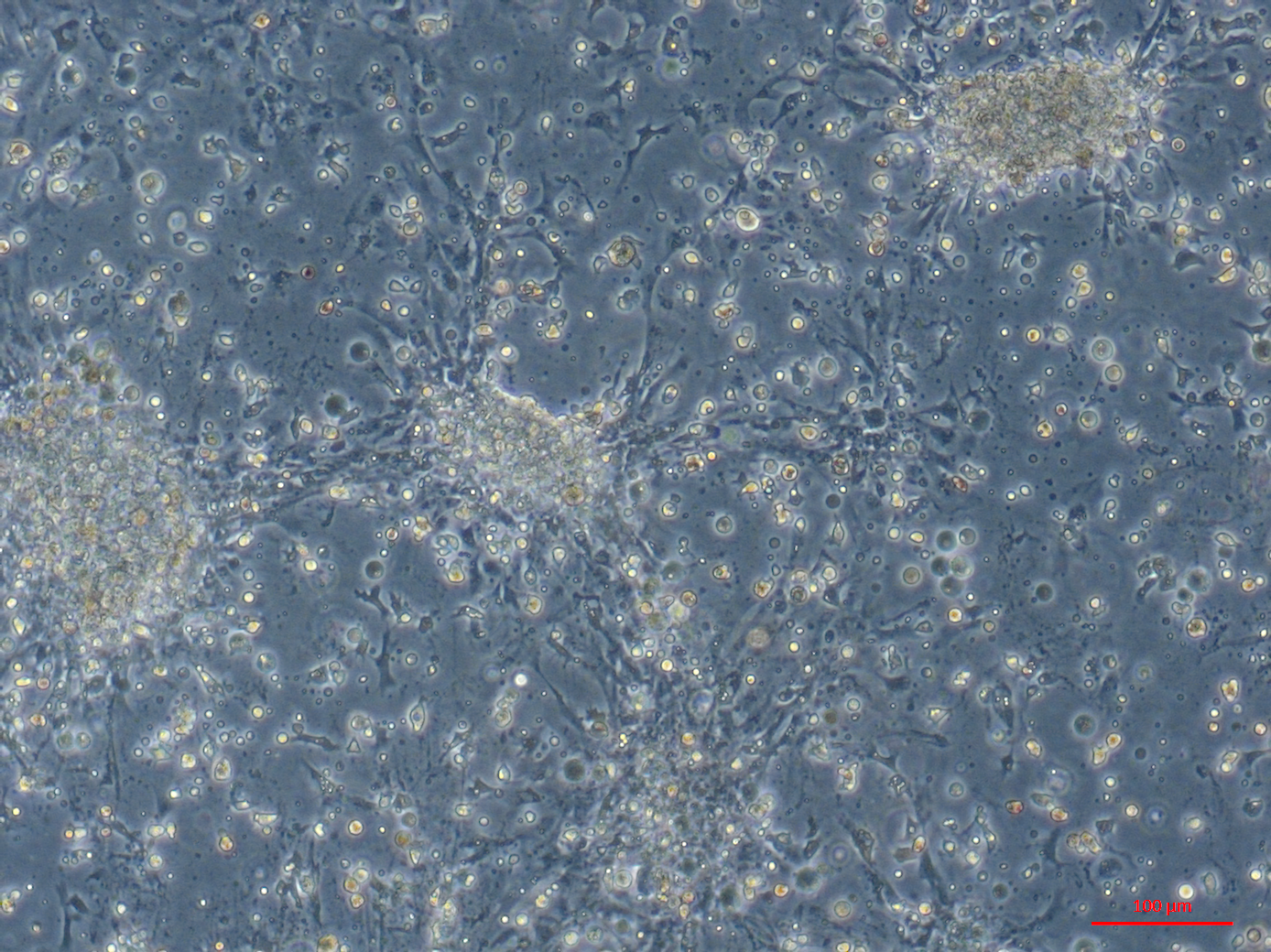

Supplement: Figure S7 — Mantle primary culture showing epithelial-like cells dissociating from aggregations of rounded cells and adhering to culture plastics, similar to heart cultures. [file peerj-08-9180-s012.png]

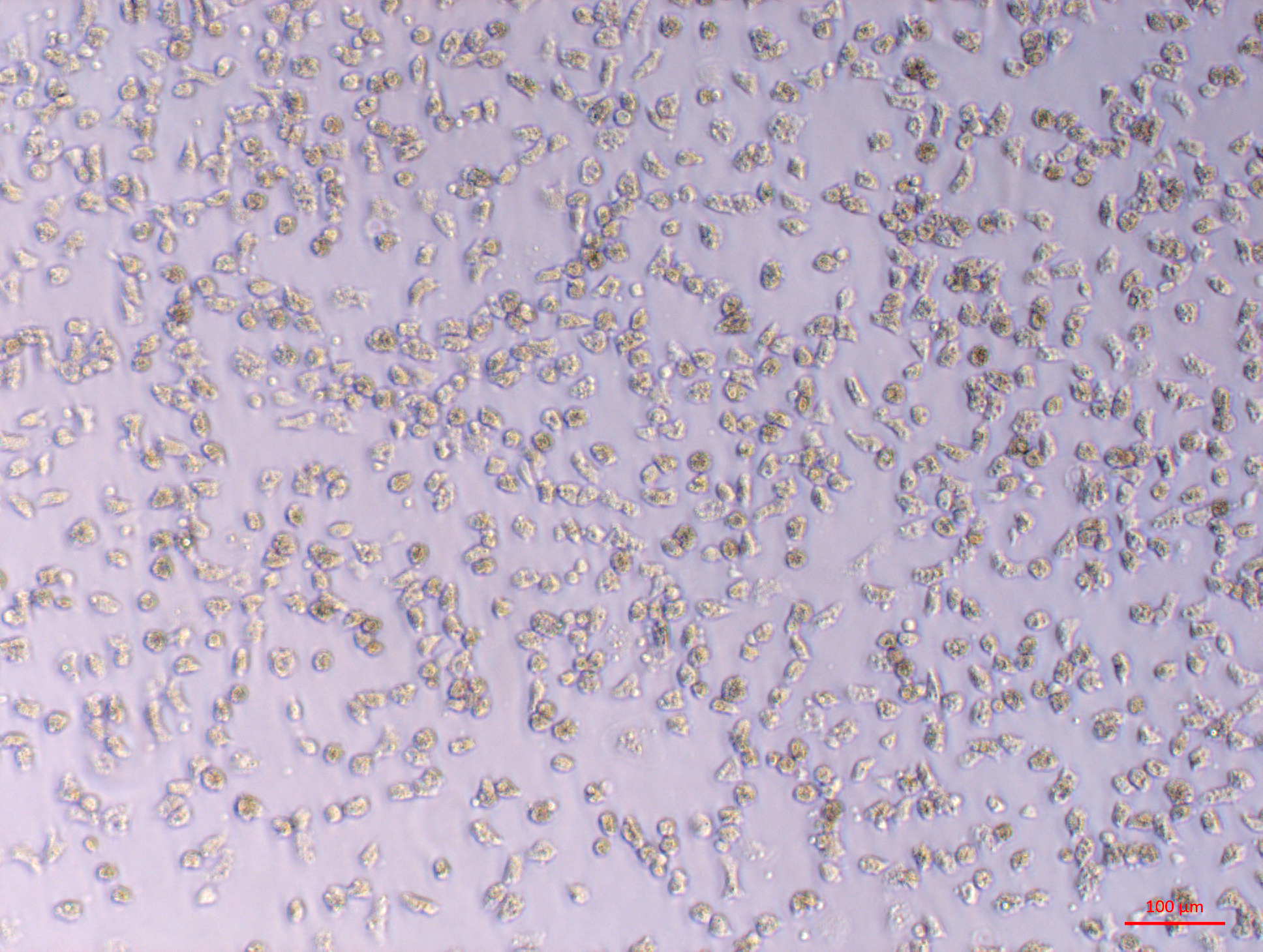

Supplement: Figure S8 — Gonad primary culture with round and fibroblast-like cells visible, 5 days post explant. [file peerj-08-9180-s013.png]

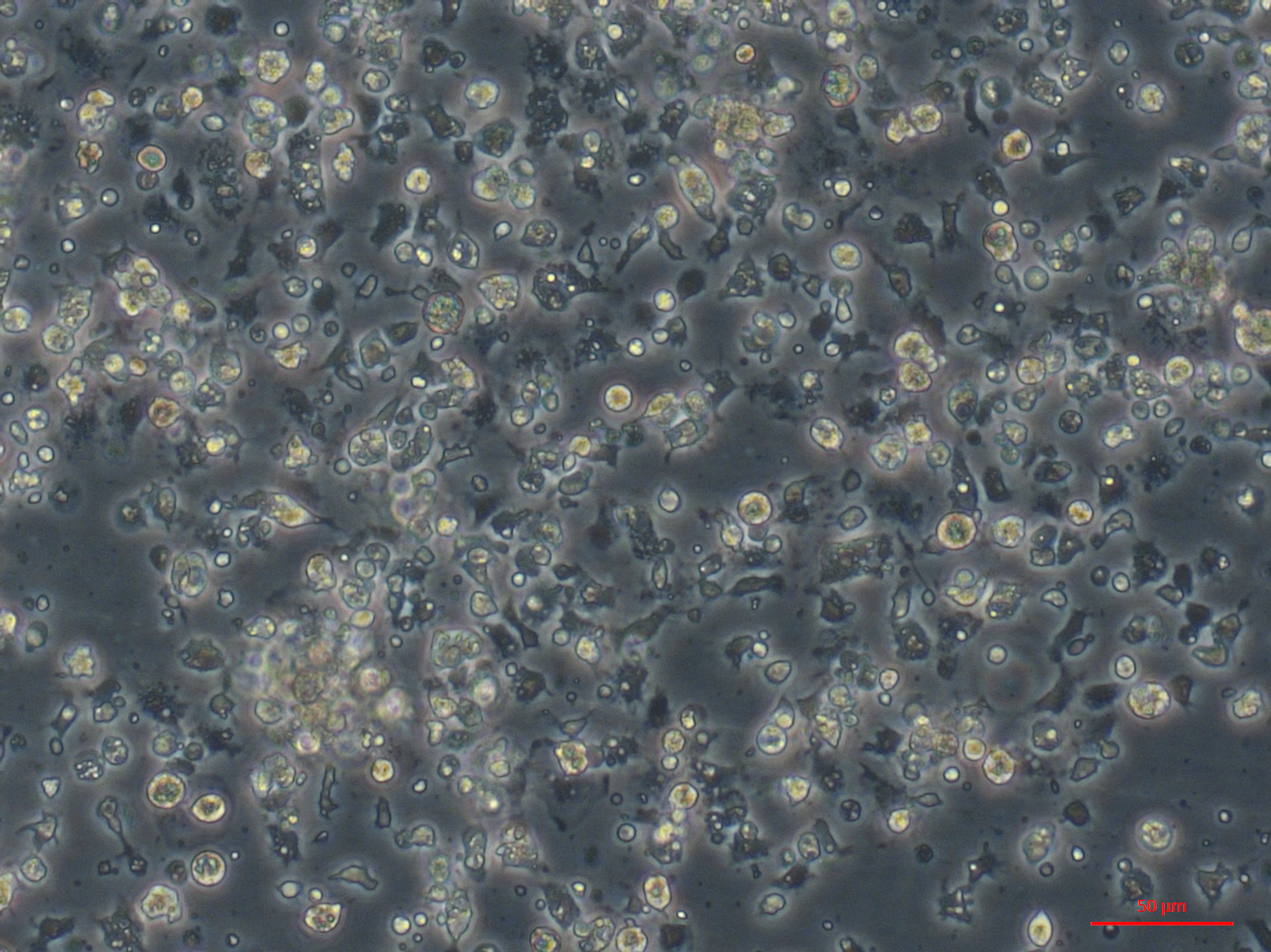

Supplement: Figure S9 — Gill primary culture with hemocytes, round and fibroblast-like cells. [file peerj-08-9180-s014.png]

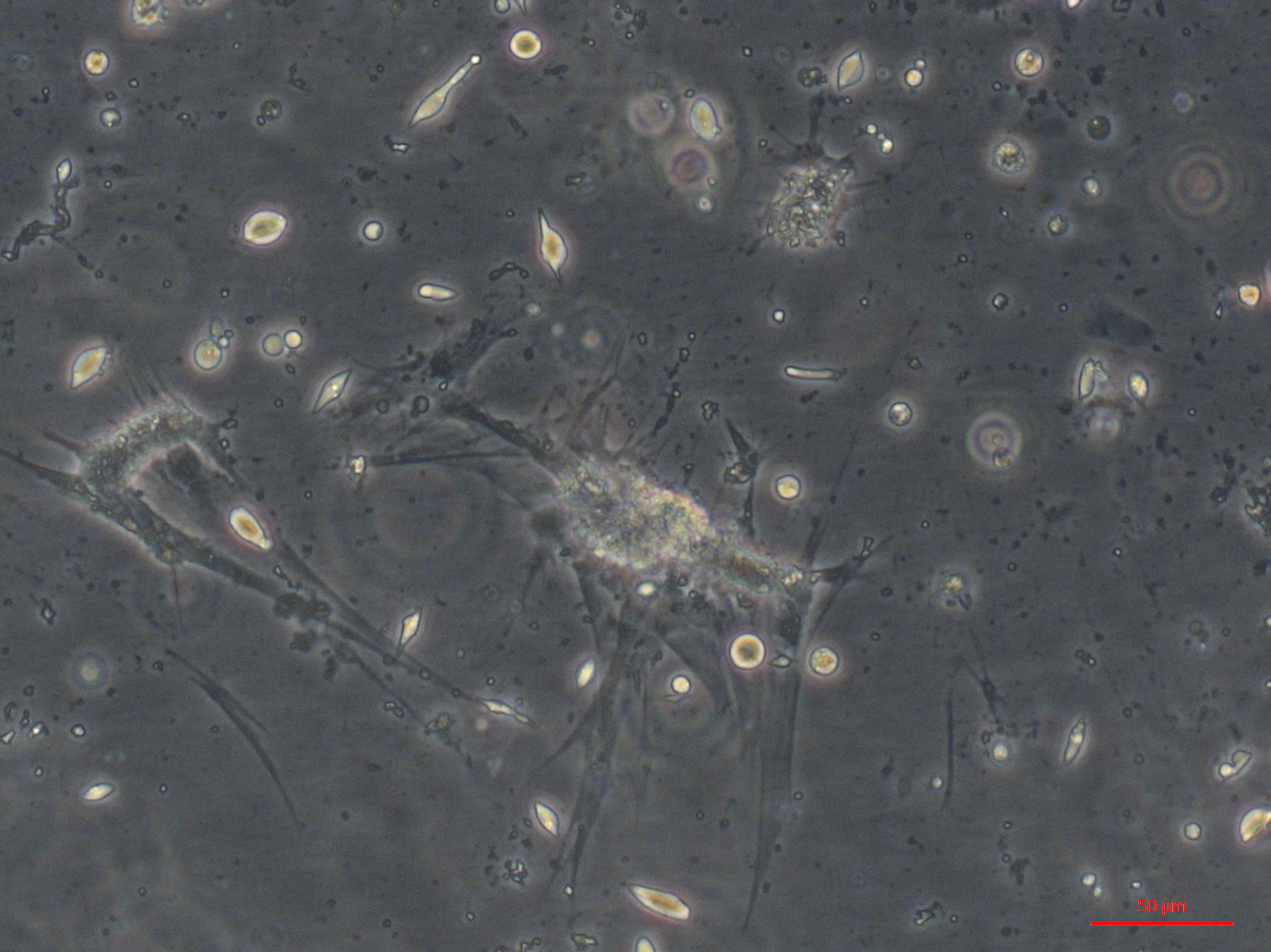

Supplement: Figure S10 — Muscle primary culture with round, spindle shaped and larger muscle-like cells adhering to the culture plastics. [file peerj-08-9180-s015.png]
